# Black solo garlic protects hepatic and renal cell function in streptozotocin-induced rats

**DOI:** 10.3389/fnut.2022.962993

**Published:** 2022-11-30

**Authors:** Saryono, Sarmoko, Desiyani Nani, Atikah Proverawati, Agis Taufik

**Affiliations:** ^1^Department of Nursing, Faculty of Health Sciences, Jenderal Soedirman University, Purwokerto, Indonesia; ^2^Department of Pharmacy, Faculty of Health Sciences, Jenderal Soedirman University, Purwokerto, Indonesia; ^3^Department of Pharmacy, Sumatera Institute of Technology, South Lampung, Indonesia; ^4^Department of Nutrition, Faculty of Health Sciences, Jenderal Soedirman University, Purwokerto, Indonesia

**Keywords:** black garlic, black solo garlic, kidney function, antioxidant, liver function

## Abstract

Black solo garlic (BSG) has been evaluated for its ability to reduce free radicals; however, the safety test on kidney and liver function has not been evaluated. This study aimed to examine the effect of brewed BSG on the liver (total protein, albumin, glutathione S-transferase/GST) and kidney (urea, creatinine, and β_*2*_-microglobulin) function in streptozotocin (STZ)-induced white rats. The experimental animals were randomly divided into six groups, each including five animals. The groups consist of the normal control group, the STZ-induced control group, the BSG treatment group with doses 6.5, 13.5, and 26 g/kg body weight, and metformin positive control. After STZ induction, the serum levels of GST, total protein, and albumin are decreased. After treatment with BSG, the serum level of GST, total protein, and albumin increased significantly (*p* < 0.05). The levels of urea, creatinine, and β_2_-microglobulin increased after STZ induction. After treatment of BSG, levels of urea, creatinine, and β_2_-microglobulin are decreased significantly (*p* < 0.05). These results suggest that BSG use is safe for the liver and kidneys of STZ-induced rats.

## Introduction

Free radicals are constantly generated in the body, both during normal metabolic processes and under pathological circumstances. Due to oxidation processes, high levels of free radicals induce cell damage, particularly in liver and kidney cells. Drugs and foods are the main sources of free radicals in the human body. Foods polluted with heavy metals frequently cause health problems (1). Oxidant chemicals from free radicals cause cell damage, weaken immunity, and change cell characteristics. These result in diminished cell performance and numerous diseases, such as diabetes mellitus, neurodegenerative disease, and cancer (2). Most of the degenerative diseases are correlated with an increased level of free radicals in the body (3).

The prevalence of degenerative diseases such as renal failure, hepatic cirrhosis, liver cancer, coronary heart disease, and type 2 diabetes mellitus increases. Multiple studies show that the high incidence of degenerative diseases is linked to dietary patterns. Foods containing xenobiotic compounds, pollutants, dyes, and preservatives are a potential source of free radicals. Unpaired electrons in the outermost orbit of free radicals render them unstable. To maintain their stability, free radical species oxidize adjacent molecules and cause damage. Cells are subject to inflammation, injury, dysfunction, and death. Kidney and liver damage can be caused directly by free radicals or indirectly by other diseases’ complications (4).

The use of chemical drugs can repair cell damage and strengthen the immune system. However, such effort is ineffective because numerous chemical compounds cause liver and kidney toxicity, as well as immunosuppression (5). Reactive oxygen and nitrogen species influence hepatocytic proteins that can cause structural and functional liver problems (6). Elevated intracellular reactive oxygen species (ROS) levels are linked to chronic renal disease (7). Certain xenobiotic chemicals are processed by the liver and eliminated by the kidneys. If a harmful substance such as streptozotocin (STZ) enters the body, it will be detoxified in the liver, conjugated, and then eliminated *via* the kidneys. To prevent harmful damage from these substances, cells require an abundance of antioxidant molecules. As an injection of STZ surpasses the threshold ability of the liver and kidneys, it will produce toxicity and inflammation in the liver and kidneys.

Some medicinal plants are rich in antioxidants and garlic (*Allium sativum* L.) being one of them. Garlic contains alliin (S-allyl cysteine sulfoxide), an unstable compound that rapidly is converted into allicin (8). There are numerous varieties of garlic, including those with multiple bulbs, and those with a single bulb, but single-bulb garlic (solo garlic) has the highest antioxidant capacity (9). All the active compounds in solo garlic are concentrated in a single clove. Garlic can be fermented into black garlic to diminish its flavor and improve digestive comfort (10).

Black garlic was obtained by heating it at 65–80°C for 20–40 days. Because the level of S-Allyl-cysteine in black garlic is much higher than in raw garlic, its antioxidant activity is stronger (11). Black garlic contains organosulfur compounds with potent antioxidant and free radical scavenger properties (12). During its aging process, the level of S-allyl-cysteine, phenolic acids, and flavonoids increases (13). Previous studies showed that the aqueous extract of black garlic possesses antioxidant and anti-inflammatory properties that can alleviate colistin-induced acute renal failure (14). The safety of BSG and its antioxidant effect on liver and kidney functions have not been tested. Therefore, the purpose of the study was to determine the effect of brewed BSG on the liver and kidney functioning of STZ-induced white rats.

## Materials and methods

### Plants

Solo garlic was selected for being nearly identical in size and defect free. Fresh solo garlic was obtained from a garlic farm in Brebes, Central Java, and authenticated in the plant taxonomy laboratory of the Faculty of Biology, Jenderal Soedirman University, Indonesia.

### Fermentation process

The fresh solo garlic was wrapped with paper tissue and placed in rice cooker-modified fermentation apparatus. The modified fermentation equipment was put to warm mode (temperatures between 60 and 80°C) and monitored for up to 21 days. Every 3 days, any changes in the condition of the garlic were observed and monitored. Any dew was dried up using paper tissue and then rewrapped with aluminum foil. After 21 days, the garlic turned black and had a chewy texture (15).

### Black solo garlic preparation

The BSG was peeled and weighed per dose before being mashed using a pestle and mortar. The fine garlic was placed into a glass cup and dissolved in hot water (200 mL per dose) at 80–90°C, agitated until well combined, and left for 15 min. The BSG was filtered to obtain the brewed water, which was then left to cool. The brewed BSG was administered to the rats using a syringe and a sonde (15).

### Preparation of animal experiments

This research was conducted upon ethical consideration from the Ethical Commission for Health Research, Faculty of Health Sciences, Jenderal Soedirman University, No: 152/EC/KEPK/VII/2020. The study employed an experimental design with pre- and post-testing and a control group.

The weight of experimental animals, 8-month male Wistar rats, was between 150 and 250 g. The animals were assigned at random into six groups, each including five animals. During the study, the animals were fed *ad libitum* BR II and distilled water. Before the study, the animals were acclimatized for 7 days. The animals were housed in 20 cm × 30 cm × 15 cm plastic box cages under a 12:12-h light/dark (L/D) cycle. The boxes were filled with 2 cm of sawdust and covered with a woven wire measuring 30.5 cm × 20.5 cm × 3.5 cm with woven area of 0.5 cm^2^. The sawdust was replaced regularly to maintain cleanliness and comfort. The room temperature was maintained at 25°C for the comfort of the animals. After the rats were adapted, induction of STZ was performed.

**FIGURE 1:**
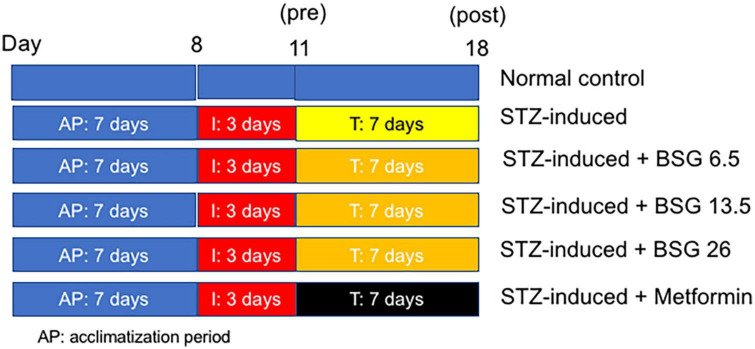
A graphical scheme showing the timeline of STZ and BSG/metformin treatment.

On the eighth day, after a 6–8-h fast, STZ was administered to induce damage of liver and kidney organs. Rats were given STZ 50 mg/kg body weight (BW) that had been dissolved in 2.5 mL of 0.05 M citrate buffer intra-peritonial (15). On Day 11, following 3 days of induction, the animals were treated with BSG extract for 14 days (Figure 1).

### Treatments

This study used six groups: a normal control group, an STZ-induced control group, BSG treatment groups with doses of 6.5, 13.5, and 26 g/kg BW, and a positive control group administered 10 mg/kg BW of metformin. The rats in the intervention group, which consisted of groups BSG 6.5 (6.5 g/kg BW dose), BSG 13 (13 g/kg BW dose), and BSG 26 (26 g/kg BW dose) were given brewed BSG in the morning and afternoon.

Black solo garlic (BSG) was peeled, weighed according to dosage, and then crushed with a pestle and mortar. BSGs were placed in a glass, and then hot water (200 mL per dose) was added. The mixture was stirred thoroughly for 15 min before being set aside. The BSG liquid mixture was filtered to obtain the steeping water, and then it was allowed to cool. The steeping was administered orally to the rats using a syringe.

### Variable measurement

Three milliliters of blood were taken from the orbital plexus using a capillary pipette. The first and last blood samples were obtained from the same location. The variables measured were urea, creatinine, β_2_-microglobulin, total protein, albumin, and glutathione S-transferase (GST). Rats were euthanized and decapitated by being put into a large bottle containing ether (16).

The urea level was determined using a UV Vis spectrophotometer with the Berthelot method at 578 nm. Creatinine was measured using a UV Vis spectrophotometer with the Jaffe kinetic method at 492 nm. Total protein was determined using a UV Vis spectrophotometer with the Biuret method at 546 nm. Albumin was quantified using a UV Vis spectrophotometer with the Bromine Cressol Green method at 578 nm.

The levels of β_2_-microglobulin and GST were determined using an ELISA kit (BT Laboratories, Shanghai) in accordance with manufacturer’s protocol. The optical density (OD) was measured using an ELISA reader (Labotrone, Germany) at 450 nm. Each level variable was determined by comparing the OD of the samples to the standard curve.

### Statistical analysis

The data were displayed as mean ± SEM. The difference between the treatment and control groups was analyzed using the one-way ANOVA test. The graphic was created using the Prism GraphPad 8.0 software (San Diego, USA). The test result was considered significant if the *p*-value was less than or equal to 0.05.

## Results

### Black solo garlic’s effect on renal function

**FIGURE 2:**
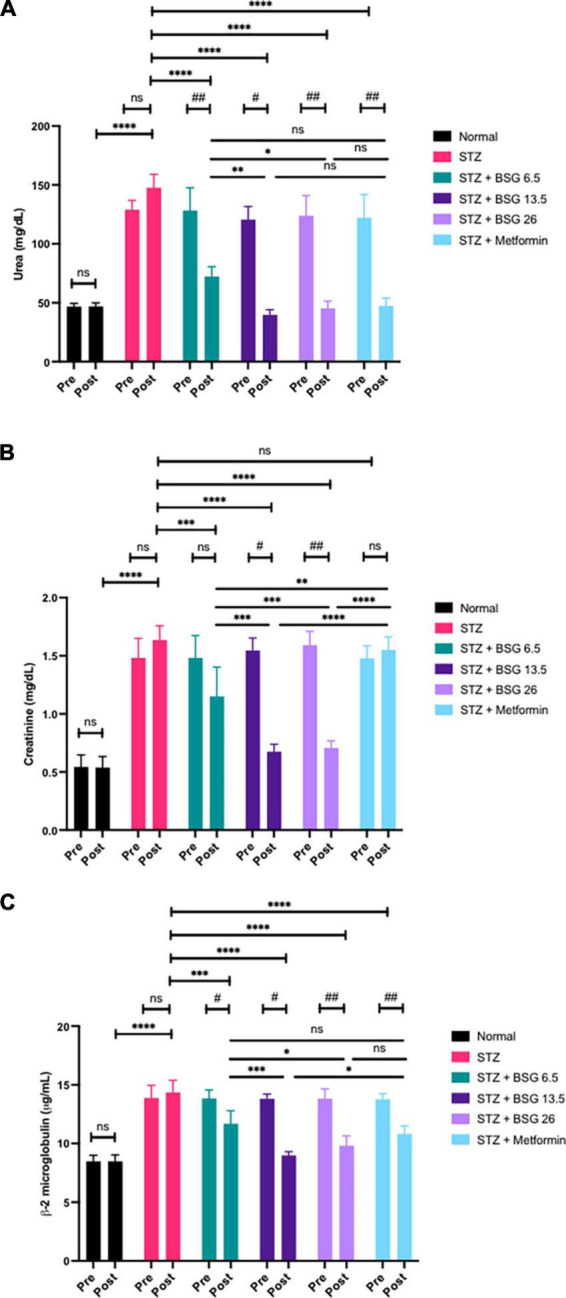
Black solo garlic protects the renal function. **(A)** Urea level, **(B)** creatinine, and **(C)** β_2_-microglobulin after treatment of black solo garlic (BSG) 6.5, 13.5, and 26 g/kg BW dose in streptozotocin (STZ)-induced rats. ****p < 0.001, ***p < 0.005, ** or ^##^*p* < 0.01, * or ^#^*p* < 0.05, ns, not significant.

The results indicate that STZ-induced rats significantly increased the levels of urea, creatinine, and β_2_-microglobulin levels (Figure 2). The administration of BSG at doses of 13.5 and 26 g/kg BW decreased urea and creatinine levels by 60% and β_2_-microglobulin levels by 30% when compared with the STZ group. Therefore, a dose of 13.5 g/kg BW of BSG is equivalent to a dose of 26 g/kg BW for ameliorating the toxic effects of STZ administration.

Induction of STZ increased the level of blood urea, creatinine, and β_2_-microglobulin than the normal group. There was no difference in the urea levels between the 13.5 g/kg BW dose of BSG, the 26 g/kg BW dose of BSG, metformin, and the normal group, showing that BSG can improve the filtration of blood by kidney cells, so that they can excrete urea again. This BSG treatment restores normal blood urea levels in rats treated with STZ.

There was no difference in creatinine levels between the BSG 13.5, BSG 26, and normal control after administration of BSG, although there was a significant difference between the metformin and BSG 6.5 group (*p* < 0.001). It appears that BSG at doses of 13.5 and 26 g/kg BW, but not 6.5 g/kg BW, repaired the kidney cells, restoring the cell’s ability to keep the normal blood creatinine levels as in the normal group. In comparison to the pre-test, the metformin group’s post-test creatinine showed no significant difference, given that metformin has a potential side effect on lactic acidosis.

Beta-2-microglobulin is a nucleated cells-produced protein. β_2_-microglobulin is filtered by the glomerulus, but it is reabsorbed. The results showed that β_2_-microglobulin levels in BSG 13.5, BSG 26, metformin, and the normal group did not differ significantly. Kidney dysfunction leads to an increase in β_2_-microglobulin. β_2_-microglobulin is a highly sensitive indicator of kidney cell damage, indicating that BSG can improve the ability of kidney cells to filter the blood effectively. Low-damage cells release a low level of β_2_-microglobulin protein. The blood β_2_-microglobulin level of rats administered brewed BSG returned to normal.

### Black solo garlic’s effect on hepatic function

**FIGURE 3:**
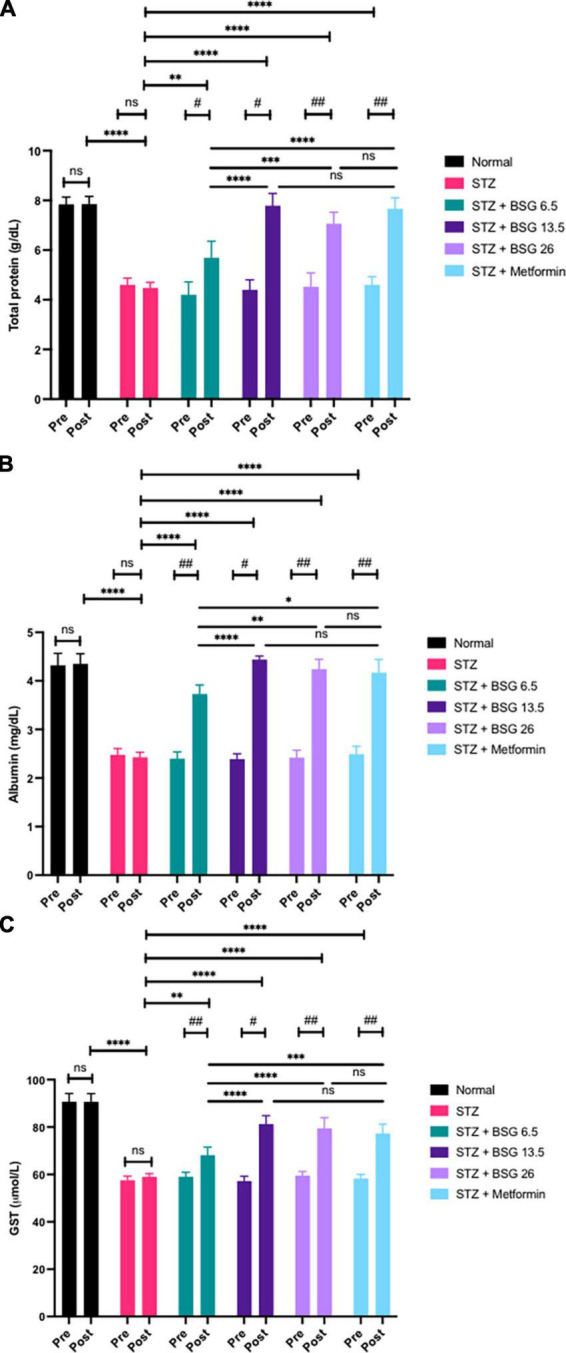
Black solo garlic protects the hepatic function. **(A)** Total protein, **(B)** albumin, and **(C)** GST level after treatment of black solo garlic (BSG) 6.5, 13.5, and 26 g/kg BW dose in streptozotocin (STZ)-induced rats. ****p < 0.001, ***p < 0.005, ** or ^##^*p* < 0.01, * or ^#^*p* < 0.05, ns, not significant.

STZ induction can have an adverse effect on the liver, impairing its function. Toxic metabolites lead to the oxidation of liver cells, resulting in liver inflammation. Damage to the liver can diminish levels of total protein, GST enzymes, and albumin. BSG at doses of 13.5 and 26 g/kg BW can restore normal levels of total protein, GST, and albumin (Figure 3).

STZ induction can compromise liver function, resulting in decreased levels of total protein, albumin, and GST when compared with the normal group (*p* < 0.001). The results showed that treatment of BSG 6.5, BSG 13.5, BSG 26, and metformin increased levels of total protein (pre vs. post), and the increased level is equal to the normal group, suggesting that BSG can restore liver cell function. Similarly, there was no difference in albumin levels between BSG 13.5, BSG 26, metformin, and the normal group, indicating that BSG can improve liver cells’ ability to synthesize protein, so allowing them to increase albumin levels. The BSG treatment restored normal blood albumin levels in rats treated with STZ.

STZ induction significantly decreased GST compared to normal controls. The results indicate that the administration of BSG can increase GST, whereas, in the STZ control, the value remains unchanged because no active compounds are administered (negative control). There was no difference in GST levels between BSG 13.5, BSG 26, metformin, and the normal group (post), suggesting the treatment of BSG can restore GST levels equal to the normal group in the STZ-induced animal model. STZ-induced liver cell injury resulted in decreased GSH production, leading to decreased GST activity. BSG can repair liver cells, restoring the normal function of liver enzymes. An increase in GSH reactivated and increased the level of GST.

## Discussion

Induction of STZ results in damage to the pancreatic beta cell (15). The cell damage leads to insulin secretion disorders, causing hyperglycemia. Hyperglycemia induces metabolic and hemodynamic changes that contribute to kidney injury. Several metabolic pathways are stimulated by hyperglycemia, including activation of protein kinase C, increased production of advanced glycosylation end products (AGEPs) and diacylglycerol, and increased ROS (17). Hemodynamic changes are mediated by the increase in angiotensin II production. Angiotensin II promotes podocyte-derived VEGF receptors (VEGFR1 and VEGFR2), suppresses nephrin expression, and induces TGF-β. Consequently, increased VEGF promotes the proliferation of cells that express vascular endothelial growth factor receptors (VEGFR-1) and VEGFR-2. Cell proliferation results in glomerular hypertrophy and kidney enlargement.

STZ induction can lead to kidney and liver cell damage due to a free radical oxidation process. High levels of ROS such as superoxide, peroxyl, and hydroxyl radicals can damage microvasculature, leading to vascular injury and organ dysfunction. ROS can also induce oxidative DNA damage, which ultimately leads to cell death (18). Oxidation on the endothelial walls of blood vessels at the glomerulus can lead to endothelial cell activation. In addition, various pro-inflammatory cytokines increase the production of neutrophils near the infection site in the glomerulus, which disrupt the filtration and excretion of urea and creatinine. This disorder causes the accumulation and elevation of urea, creatinine, and β_2_-microglobulin in the body. Urea and creatinine are toxic in high concentrations in the body (19), while β_2_-microglobulin is secreted at a constant rate by all nucleated cells. The rise of these indicators indicated a renal disease.

The liver is an important organ for metabolism, biomolecular synthesis, and detoxification (20). Induction of STZ results in liver inflammation and impairment of the gluconeogenesis pathway. This liver damage decreases the generation of GST, and an essential enzyme involved in both the protection of the body from oxidative stress and the detoxification process. Additionally, the liver’s ability to decrease protein generation results in a fall in total protein and blood albumin levels. These parameters are important biomarkers to assess liver health. Under oxidative stress conditions, antioxidant enzymes such as SOD, GPX, and catalase are decreased. Bioactive components in BSG can function as antioxidants. Therefore, they can help stop the chain reaction of free radicals, so that liver and kidney cells gradually improve as indicated by total protein, albumin, and GST level.

The antioxidant action of black garlic can decrease AGEP generation by reducing lipid peroxidation and indirectly increasing NO synthesis (21). Organosulfur component and flavonoid content of black garlic are antiglycation and potent antioxidants which can repair liver and kidney cells by increasing antioxidant enzymes’ activity, such as catalase, superoxide dismutase, and glutathione peroxidase (22, 23). Previous study also showed that black garlic extract can reduce oxidative stress in kidney cells, hence reducing inflammation (14).

Black garlic contains S-allyl cysteine (SAC), which has higher bioactivity as an antidiabetic, antioxidant, and anti-inflammatory than regular garlic (24–26). Inflammation results in the production of pro-inflammatory mediators (27). Black garlic administration can suppress pro-inflammatory mediators, hence, reducing tissue damage during inflammation (28). SAC, S-allyl mercaptocysteine, and allicin contained in black garlic are potent antioxidant chemicals (27). SAC is able to eliminate superoxide anions, hydrogen peroxide, hydroxyl radicals, peroxynitrite radicals, and peroxyl radicals generated by neural cells, as well as hypochlorous acid and singlet oxygen generated by microglial cells (29).

The chemical constituents of BSG are equal to those of regular garlic, with the addition of alliin (411.4 mg/mL) and allicin (268.2 mg/mL (30). The constituents may protect liver and kidney cell function in STZ-induced rats by restoring the normal level of urea, creatinine, β2-microglobulin, GST, total protein, and albumin.

## Conclusion

BSG protects liver and kidney cell function in STZ-induced rats by restoring the normal level of urea, creatinine, β2-microglobulin, GST, total protein, and albumin. This action may be mediated by the high-antioxidant content of BSG’s constituents; STZ-induced kidney and liver damage is restored.

## Data availability statement

The original contributions presented in this study are included in the article/supplementary material, further inquiries can be directed to the corresponding author.

## Ethics statement

The animal study was reviewed and approved by the Komisi Etik Penelitian Kesehatan, Jenderal Soedirman University.

## Author contributions

Saryono and AP contributed to the conceptualization and project administration. Saryono, DN, AP, AT, and Sarmoko contributed to the methodology and investigation. AT contributed to the validation. Saryono and Sarmoko contributed to the formal analysis, funding acquisition, and writing of review and editing. Saryono, DN, and AP contributed to the writing of original draft preparation. Saryono and DN contributed to the supervision. All authors contributed to the article and approved the submitted version.
